# The Influence of Height‐Adjusted Nutritional Status Assessment on Complications Following Growing‐Rod Surgery for Early‐Onset Scoliosis

**DOI:** 10.1111/os.70461

**Published:** 2026-11-06

**Authors:** Yangpu Zhang, Haoshuang Geng, Jianqiang Wang, Yong Hai

**Affiliations:** ^1^ Department of Orthopedic Surgery Beijing Chaoyang Hospital, Capital Medical University of China Beijing China; ^2^ Laboratory for Clinical Medicine Capital Medical University of China Beijing China

**Keywords:** complication, early‐onset scoliosis, growing‐rod surgery, malnutrition

## Abstract

**Objectives:**

Accurate nutritional assessment in children with early‐onset scoliosis (EOS) is challenging because spinal deformity reduces measured standing height and may distort BMI‐for‐age classification. This study aimed to evaluate, using a longitudinal design, height‐adjusted nutritional status before each growing‐rod procedure throughout the treatment course and to investigate its association with postoperative complications in patients with EOS.

**Methods:**

A retrospective cohort study included 53 patients with EOS who underwent 86 growing‐rod procedures between January 2016 and January 2023, with each procedure serving as the unit of analysis. Nutritional status was reassessed before each analyzed procedure using BMI calculated from height corrected by the Ylikoski formula and was linked to the postoperative outcome of the corresponding procedure. Generalized estimating equations (GEE) were used to evaluate factors associated with postoperative complications while accounting for within‐patient clustering.

**Results:**

A total of 14 procedures resulted in postoperative complications, yielding an overall complication rate of 16.3% (14/86), with 15 specific complication events recorded. After height‐adjusted BMI correction, 46 procedures were classified into the malnourished group and 40 into the normal nutrition group. The complication rate was significantly higher in the postadjusted malnourished group than in the normal nutrition group (23.9% vs. 7.5%, *p* = 0.042). Multivariable analysis using GEE revealed that undergoing ≥ 3 surgeries (OR = 4.614, 95% CI: 1.133–18.786, *p* = 0.033), and postadjusted malnutrition (OR = 3.701, 95% CI: 1.006–13.613, *p* = 0.049) were associated with increased risk of postoperative complications. However, after the Mancl–DeRouen small‐sample correction, the association between height‐adjusted malnutrition and complications was no longer statistically significant (OR = 4.21, 95% CI: 0.38–46.12, *p* = 0.239).

**Conclusions:**

Height‐adjusted malnutrition and repeated surgeries (≥ 3) were associated with increased postoperative complications after growing‐rod surgery in EOS patients. However, larger cohorts are needed to confirm these findings. The procedure‐specific application of height‐adjusted BMI provides an alternative approach to longitudinal nutritional assessment during repeated growing‐rod treatment. Timely intervention should be implemented for patients with poorer nutritional status.

## Graphical abstract

Adjusting BMI using the Ylikoski formula provides a more accurate nutritional assessment for early‐onset scoliosis patients. Preoperative malnutrition, and undergoing three or more surgeries are risk factors significantly associated with increased postoperative complications following traditional growing‐rod surgery.

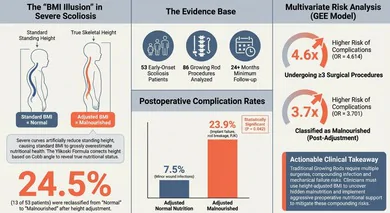

**Abbreviations**
AUC: area under the curve
BMI: body mass index
CI: confidence interval
CS: congenital scoliosis
DJK: distal junctional kyphosis
EOS: early‐onset scoliosis
IQR: interquartile range
IS: idiopathic scoliosis
PJK: proximal junctional kyphosis
ROC: receiver operating characteristic
SD: standard deviation

## 1 Introduction

Early‐onset scoliosis (EOS) refers to a spectrum of spinal deformities diagnosed at or before 9 years of age. Based on etiology, EOS can be classified into congenital, neuromuscular, idiopathic, and syndromic types. While the overall global incidence of EOS remains uncertain, evidence from a Japanese multicenter study indicated annual incidence of 0.019% [1]. EOS is often characterized by early onset of the disease and rapid progression [2, 3], while the spine is still skeletally immature, thereby requiring treatments that correct deformity while preserving spinal growth. Conservative approaches, such as bracing or casting, may be an adequate intervention for children with mild curves. However, severe cases or those unresponsive to conservative management often require surgical intervention, commonly employing various expandable devices. These include traditional growing rods (TGR), vertical expandable prosthetic titanium ribs (VEPTR), and magnetically controlled growing rods (MCGR) [4].

Among these, the growing‐rod technique has emerged as the primary nonfusion surgical option for EOS patients [5]. This method involves the placement of spinal rods with subsequent periodic lengthening procedures, enabling deformity correction while supporting continued spinal growth [6, 7].

Despite its effectiveness, complications are frequent due to the patients' young age and frequent surgical lengthening [8]. Complications related to the growing rod technique encompass internal fixation‐related complications, spine morphology‐related complications, and general complications. Kabirian et al. [9] reported implant‐related complications in 52.4% of patients and deep infections in 11.2%. The incidence of proximal junctional kyphosis (PJK) has been reported to reach 37.5%, which commonly results in the need for revision surgery [10, 11, 12]. Previous studies have reported that younger age at initial implantation and a greater number of surgical procedures are risk factors for complications associated with growing rod surgery [10, 13]. Nutritional status also plays a critical role in surgical outcomes, as severe scoliosis often compromises circulatory, respiratory, and gastrointestinal development [14], thereby aggravating malnutrition in EOS patients [15]. Furthermore, high‐altitude hypoxia has been shown to exacerbate nutritional deficiencies, thereby contributing to an increased risk of postoperative complications [16]. Although preoperative nutritional status is recognized as an important risk factor for surgical complications [17, 18], its specific influence on outcomes following growing‐rod surgery in EOS patients remains insufficiently investigated.

Given the repeated nature of traditional growing‐rod treatment, nutritional status may vary throughout the treatment course. Therefore, this study aimed to longitudinally evaluate height‐adjusted nutritional status before each analyzed growing‐rod procedure and to investigate its association with postoperative complications.

## 2 Methods

A retrospective cohort study was conducted on EOS patients undergoing growing‐rod surgery at our hospital from January 2016 to January 2023. The inclusion criteria were: (1) Diagnosis of EOS; (2) Treatment with growing‐rod surgery; (3) A minimum follow‐up of 24 months. Exclusion criteria were: (1) Concomitant cancer, tuberculosis, or chronic conditions affecting nutrition; (2) A history of non–growing‐rod spinal surgery; (3) Age > 9 years at the time of the index surgery; (4) Neuromuscular and syndromic types of scoliosis were excluded (Figure 1). The study adhered to the Declaration of Helsinki and was approved by our hospital's ethics committee (approval number 2022‐KE‐208), with informed consent obtained.

**FIGURE 1:**
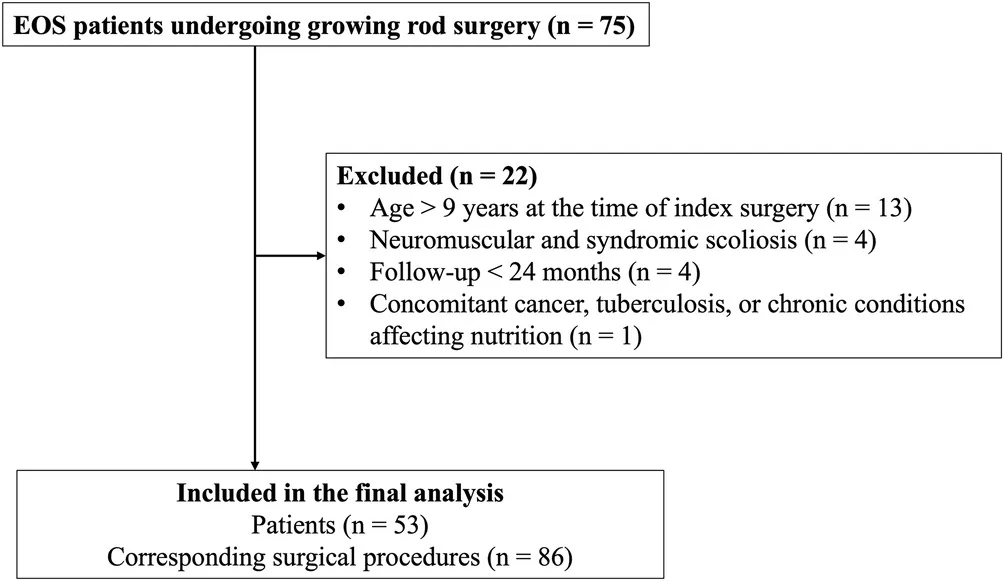
Flowchart of patient enrollment and the selection process.

### 2.1 Surgery Protocol

Surgery was indicated for a major curve > 50° and failed conservative treatments. Anchorage points were selected under fluoroscopic guidance, followed by limited periosteal dissection and pedicle screw insertion. Prebent titanium rods were attached submuscularly, with dominos connecting ipsilateral rods in the thoracolumbar segment. Moderate distraction was applied, reserving rod length for future adjustments. Unilateral rods were used on the concave side if convex soft tissue was underdeveloped. Due to national conditions, the interval between growth rod lengthening was set at 8–12 months to reduce medical expenses, minimize the number of distractions, decrease the frequency of general anesthesia and surgical trauma, and to accommodate the inconvenience of high‐altitude regions. Only the domino connectors beneath the deep fascia were exposed during each lengthening. Both the initial surgery and subsequent expansion procedures were performed under monitoring.

The indications for stopping the growing‐rod treatment were as follows: (1) Complete skeletal maturation (Risser sign ≥ Grade 4); (2) Inability to achieve further distraction due to spontaneous fusion after spinal surgery; (3) Repeated occurrence of internal fixation complications (≥ three times) requiring early termination of growing‐rod treatment.

### 2.2 Data Collection

Data collected included demographics, spinal deformity traits, radiological parameters (major coronal curves, coronal/sagittal balance, thoracic kyphosis), nutritional status (BMI, total protein, albumin), complications, and surgical frequency. Surgical frequency ≥ 3 represented the third or subsequent procedure. All radiological parameters were independently measured by two spine surgeons using the PACS system. The inter‐rater reliability was excellent (ICC = 0.94), and the mean of the two measurements was used in the final statistical analysis. Nutritional parameters (height, weight, BMI) were recorded within 24 h of admission, alongside biochemical tests. Follow‐up lasted at least 2 years, and each hospitalization was analyzed separately for nutritional assessment.

### 2.3 Nutritional Parameters

Within 24 h of each admission, the patients were measured for preoperative height and weight while barefoot, fasting, and wearing hospital gowns. Based on these measurements, the preoperative body mass index (BMI) was calculated using the formula kg/m^2^, rounded to one decimal place. Biochemical testing conducted within the first 24 h of admission provided laboratory indicators such as total protein and albumin before surgery.

### 2.4 BMI Adjustment

A height‐adjusted formula for scoliosis known as the Ylikoski method [19] was employed in this study: Δ*Y* = 0.0062*X* + 0.0024*X* ^2^ (Δ*Y* represents the corrected height value, *X* denotes the Cobb angle in degrees, so the corrected height *Y* is equal to the measured height *Y*1 plus Δ*Y*). This correction enabled adjustment of the preoperative BMI. Due to the spinal deformity in EOS patients, the measured standing height is often lower than the actual skeletal height, which may lead to an overestimation of BMI. To address this, we applied the height‐correction model proposed by Ylikoski [19] to estimate the loss of body height caused by the spinal curvature. For each procedure, we converted BMI into a sex‐ and age‐specific BMI‐for‐age Z‐score (BAZ) according to the WHO growth reference for children and adolescents aged 5–19 years, using age in completed months. A BAZ < −2 was classified as low BMI‐for‐age (malnutrition), including thinness (−3 ≤ BAZ < −2) and severe thinness (BAZ < −3) [20].

### 2.5 Complications

Various types of complications encountered during growing‐rod treatment were recorded and classified into three categories: internal fixation‐related complications, alignment complications, and general complications. Internal fixation‐related complications include hook dislocation, screw pullout, and rod breakage. Alignment complications include coronal or sagittal plane imbalance, junctional kyphosis (proximal or distal), curve progression (above or below the instrumented levels), and curve progression after surgery. General complications included postoperative wound infection and impaired wound healing. Postoperative complications were recorded from the date of each analyzed procedure until the next growing‐rod procedure and were attributed to the corresponding procedure.

### 2.6 Statistical Analyses

Statistical analyses were performed using SPSS software (version 20.0; SPSS Inc., Chicago, IL, USA) and R software (version 4.4.1; Vienna, Austria). Continuous variables were tested for normality and expressed as mean ± standard deviation (SD) if normally distributed, or as median (interquartile range, IQR) if not. Categorical variables were presented as frequencies and percentages.

To appropriately account for pseudoreplication bias arising from analyzing 86 surgical procedures from 53 patients, generalized estimating equations (GEE) with a binary logistic model were used. A univariate GEE analysis was first conducted to screen for potential risk factors associated with the occurrence of postoperative complications. Subsequently, to strictly adhere to the events‐per‐variable (EPV) rule and prevent model overfitting given the limited number of complication events, our final multivariate GEE model was restricted to a maximum of two independent predictors from the univariate screening. We additionally performed a sensitivity analysis of the final multivariable GEE model using the Mancl–DeRouen small‐sample correction for the sandwich variance estimator. A two‐sided *p*‐value < 0.05 was considered statistically significant.

## 3 Results

### 3.1 Patient and Procedure Characteristics

This study included 53 patients with EOS who underwent growing‐rod surgery between January 2016 and January 2023, totaling 86 surgeries, with a mean distraction frequency of 2.74 ± 0.89 times (range: 1–6). The cohort comprised 28 males and 25 females, with a mean age of 7.92 ± 0.99 years (range: 6–9), and included 17 congenital and 36 idiopathic scoliosis cases.

### 3.2 Height‐Adjusted Nutritional Classification

Using the Ylikoski formula, the corrected BMI (14.87 ± 1.68 kg/m^2^) was significantly lower than the precorrected BMI (15.38 ± 1.70 kg/m^2^) (*p* < 0.001), resulting in changes in nutritional status. For descriptive purposes, at the patient level (*n* = 53), the preadjusted BMI assessment classified 16 patients as malnourished and 37 as normal. After height‐adjusted BMI correction using the Ylikoski formula, 29 patients were reclassified as malnourished and 24 as normal. At the procedure level (*n* = 86), which serves as the unit of analysis, the cohort was divided into the postadjusted malnutrition group (46 cases) and the postadjusted normal nutrition group (40 cases). Baseline characteristics were compared between the two groups using GEE to account for within‐patient clustering. Older age and larger preoperative main curve Cobb angle were associated with increased likelihood of postadjusted malnutrition; other baseline characteristics showed no significant differences (Table 1). Given the potential relationship between Cobb angle and height‐adjusted nutritional classification, an additional multivariable GEE sensitivity analysis incorporating preoperative Cobb angle was performed. Height‐adjusted malnutrition remained associated with postoperative complications (OR = 3.94, 95% CI: 1.05–14.77, *p* = 0.041), whereas Cobb angle was not significant (*p* = 0.763) (Supplement Table 1). Although the point estimate remained similar after inclusion of Cobb angle, this analysis could only partially address the potential confounding effect of curve severity and could not establish that the nutritional exposure was independent of deformity severity.

**TABLE 1:** Comparison of baseline characteristics between normal nutrition and adjusted malnutrition groups using generalized estimating equations.

	Normal nutrition group	Malnutrition group	OR	95% CI	*p*
Age^a^	10.05 ± 2.01	11.65 ± 2.29	1.23	1.02–1.49	**0.033**
Gender			0.76	0.29–1.98	0.571
Male	25 (51.0%)	24 (49.0%)			
Female	15 (40.5%)	22 (59.5%)			
Etiology			0.53	0.17–1.68	0.281
IS	33 (50.8%)	32 (49.2%)			
CS	7 (33.3%)	14 (66.7%)			
Surgery frequency			1.48	0.65–3.36	0.353
< 3	16 (45.7%)	19 (54.3%)			
≥ 3	24 (47.1%)	27 (52.9%)			
Prealbumin (mg/dL)	19.05 ± 3.31	18.37 ± 5.07	0.98	0.89–1.08	0.674
Albumin (g/L)	41.59 ± 2.39	41.93 ± 3.82	1.06	0.92–1.21	0.444
Total protein (g/L)	65.52 ± 11.42	66.85 ± 5.35	1.01	0.987–1.03	0.422
Preoperative main curve (°)	54.79 ± 14.68	64.11 ± 16.11	1.04	1.005–1.07	**0.022**

*Note:* Bold values indicate statistically significant results (*p* < 0.05).

^a^ The patient's age at the time of each surgical procedure.

### 3.3 Postoperative Complications

Among the 86 surgical procedures, at least one complication occurred following 14 procedures, resulting in an overall complication rate of 16.28%. It is worth noting that a single surgical procedure could result in multiple types of complications. Consequently, a total of 15 specific complication events were recorded across these 14 procedures, which included six postoperative infections, eight fixation‐related complications, and one spine morphology‐related complication. The distribution of specific postoperative complications according to height‐adjusted nutritional status is shown in Table 2. When comparing the incidence of complications between the preadjusted normal nutrition group and the malnutrition group, procedures performed in malnourished patients showed a higher complication rate (25.9% vs. 11.9%), although the difference was not statistically significant (*p* = 0.164). The observed complication rate was higher in the height‐adjusted malnutrition group than in the normal nutrition group (23.9% vs. 7.5%). In the univariable logistic GEE analysis accounting for within‐patient clustering, height‐adjusted malnutrition was associated with postoperative complications (OR = 3.062, 95% CI: 1.044–8.981; Wald *p* = 0.042) (Table 3, Figure 2).

**TABLE 2:** Distribution of postoperative complications according to postadjusted nutritional status in EOS surgical procedures.

	Normal nutrition group (*N* = 40)	Malnutrition group (*N* = 46)
Internal fixation‐related complications		
Rod breakage	0 (0%)	8 (17.4%)
General complications		
Wound infection	3 (7.5%)	3 (6.5%)
Alignment complications		
Proximal junctional kyphosis	0 (0%)	1 (2.2%)

**TABLE 3:** Comparison of clinical characteristics and univariate generalized estimating equation analysis for complications.

Parameter	Complication group	Noncomplication group	OR	95% CI	*p*
Age^a^	11.00 ± 1.96	11.14 ± 2.29	0.949	0.780–1.155	0.600
Gender			0.837	0.233–3.008	0.785
Male	9 (18.4%)	40 (81.6%)			
Female	5 (13.5%)	32 (86.5%)			
Preoperative main curve (°)	59.48 ± 19.70	59.83 ± 15.42	1.002	0.964–1.042	0.926
Preoperative TK (°)	56.45 ± 24.56	52.38 ± 19.47	1.007	0.977–1.039	0.647
Preoperative LL (°)	35.94 ± 18.64	43.04 ± 14.62	0.973	0.938–1.009	0.134
C7PL‐CSVL (cm)	16.18 ± 17.51	13.50 ± 21.54	0.998	0.977–1.020	0.889
SVA (mm)	16.19 ± 13.81	16.66 ± 19.81	1.000	0.980–1.021	0.993
Etiology			3.917	0.986–15.564	0.052
IS	7 (11%)	58 (89%)			
CS	7 (33%)	14 (67%)			
Surgery frequency			3.378	1.233–9.257	**0.018**
< 3 times	2 (5.7%)	33 (94.3%)			
3 times	12 (24%)	39 (76%)			
Adjusted nutritional status			3.062	1.044–8.981	**0.042**
Normal nutrition	3 (7.5%)	37 (92.5%)			
Malnutrition	11 (24%)	35 (76%)			
Albumin (g/L)	41.31 ± 2.81	41.86 ± 3.31	0.930	0.815–1.061	0.281
Total protein (g/L)	64.15 ± 6.09	66.64 ± 9.08	0.974	0.932–1.018	0.251

*Note:* Bold values indicate statistically significant results (*p* < 0.05).

^a^ The patient's age at the time of each surgical procedure.

**FIGURE 2:**
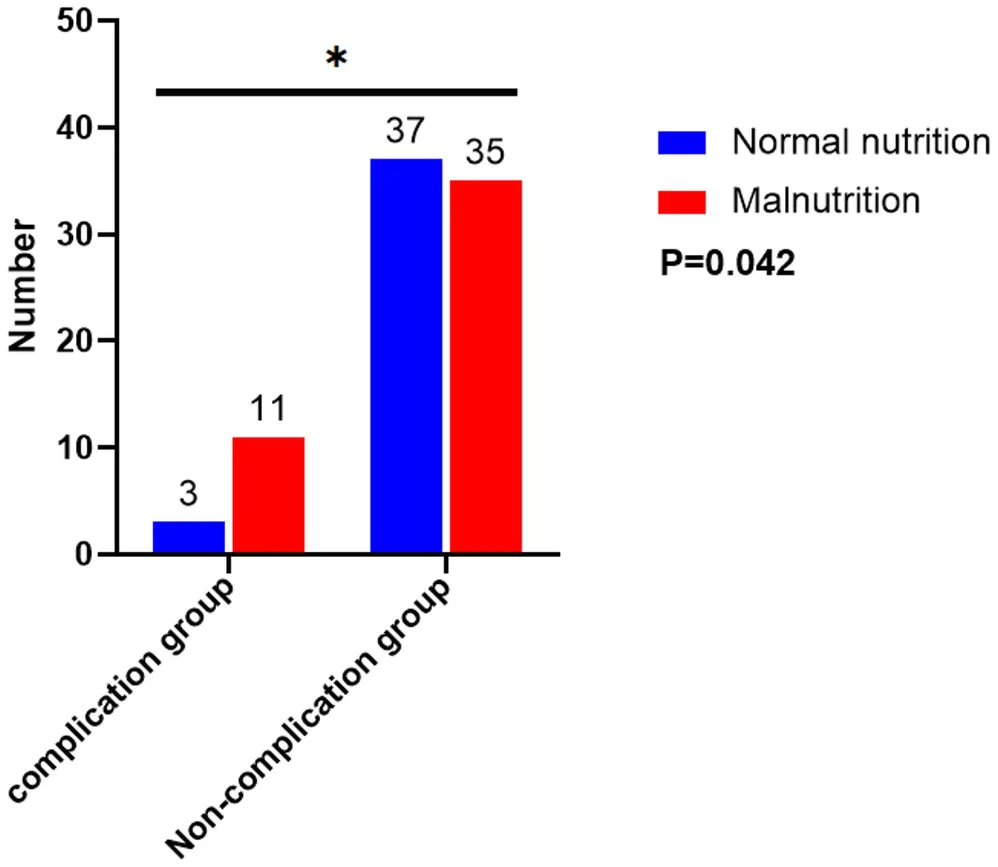
Comparison of postoperative complication rates between surgical procedures according to height‐adjusted nutritional status; *p* = 0.042 was derived from the Wald test in the univariable logistic GEE model.

### 3.4 GEE and Sensitivity Analyses

When comparing patients with and without complications, those who developed complications exhibited lower levels of albumin and total protein (41.3 vs. 41.8 g/L and 64.1 vs. 67.5 g/L, respectively), which did not reach statistical significance. A univariate GEE analysis was conducted to identify potential factors influencing complications following growing‐rod surgery (Table 3). Given that complications occurred after only 14 procedures, the multivariable model was deliberately restricted to two predictors to reduce overparameterization and model instability. Height‐adjusted nutritional status was retained because it was the primary exposure of interest, while surgical frequency was retained because of its clinical relevance to repeated growing‐rod procedures and its association in the univariable GEE analysis. Univariable *p*‐values were not used as an automatic stepwise‐selection criterion, and the remaining variables were not entered because the limited number of outcome events did not support a larger multivariable model. Further multivariate GEE analysis identified these factors as independent predictors of complications following growing‐rod surgery (Table 4). However, after the Mancl–DeRouen small‐sample correction, the association between height‐adjusted malnutrition and complications remained directionally consistent but was no longer statistically significant (OR = 4.21, 95% CI: 0.38–46.12, *p* = 0.239). The AUC for the multivariate regression analysis was 0.749 (Figure 3).

**TABLE 4:** Multivariate generalized estimating equation analysis of factors for postoperative complications.

Parameters	OR	95% CI	*p*
Surgery frequency	4.614	1.133–18.786	**0.033**
Adjusted nutritional status	3.701	1.006–13.613	**0.049**

*Note:* Bold values indicate statistically significant results (*p* < 0.05).

**FIGURE 3:**
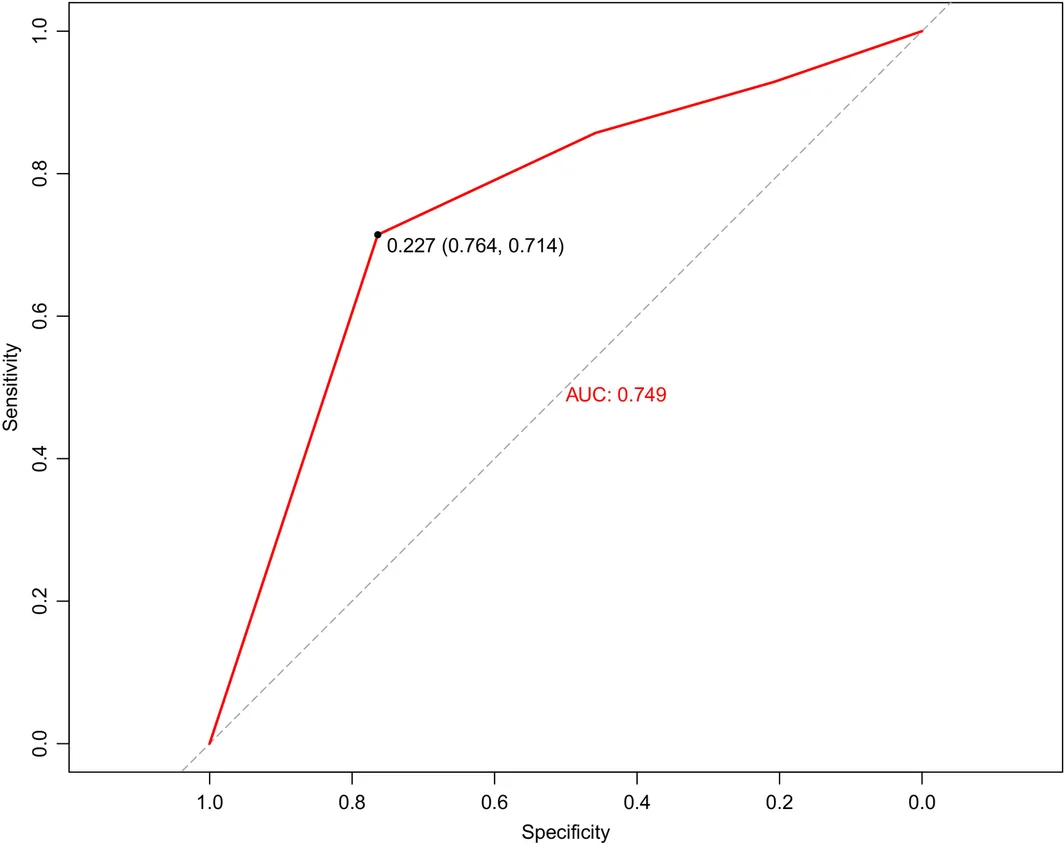
ROC curve for the multivariate model of complications after growing rod surgery in EOS patients.

## 4 Discussion

### 4.1 Principal Findings

A key feature of the present study is the longitudinal, procedure‐specific assessment of nutritional status during repeated traditional growing‐rod treatment. Nutritional status was reassessed before each analyzed procedure and linked to the postoperative outcome of the corresponding surgery, allowing evaluation of nutritional status throughout the prolonged treatment course. In the conventional multivariable GEE model, malnutrition defined by height‐adjusted BMI using the Ylikoski formula was associated with postoperative complications. However, this association was no longer statistically significant after the Mancl–DeRouen small‐sample correction (*p* = 0.239). Using GEE to control for repeated surgeries within the same patient, we observed an association between postadjusted nutritional status, surgery frequency, and postoperative complications. These results show that adjusting BMI for spinal deformity is necessary for accurate nutritional assessment and suggest that early nutritional support may improve surgical outcomes. However, larger studies are needed to clarify the relationship between nutritional status and postoperative complications.

### 4.2 Postoperative Complications During Growing‐Rod Treatment

Complications from growing‐rod surgery include internal fixation issues (e.g., rod breakage), spine morphology problems (e.g., PJK, DJK), and general issues like wound infection. Past studies report complication rates of 23.3%–66%, with fixation‐related issues comprising 76.9% [21, 22, 23]. In this study, a 16.28% overall complication rate was reported, and 57.14% of them were fixation‐related complications (8/14), lower than reports of prior research. This difference arises from treating each distraction as a separate case, analyzing nutritional status and complications over time. While this increases sample size and lowers the overall rate, other studies count complications across the entire adjustment cycle, yielding higher rates.

### 4.3 Interpretation of Height‐Adjusted Nutritional Assessment

Although BMI is a standard indicator of nutritional status, its reliability in EOS patients is limited due to height reduction. This study applied the Ylikoski formula to adjust height and BMI, better reflecting nutritional status [19, 24]. The results revealed discrepancies in BMI and nutritional grouping pre‐ and postadjustment, leading to the reclassification of certain patients from “normal” to malnourished. In our study, 13 cases (24.53%) were reclassified from the “normal” group to the malnourished group after adjustment. This improved method is vital in clinical practice to identify malnourished EOS patients overlooked for nutritional support, ensuring timely intervention.

In this study, we analyzed EOS patients with different nutritional statuses after adjustment and found that patients with postadjusted malnutrition had a higher incidence of postoperative complications. Previous studies have investigated the association between nutritional status and postoperative complications in patients with spinal deformities. Adogwa et al. [25] found that nutritional status can affect the outcomes of spinal deformity surgery, and patients with preoperative malnutrition have a higher risk of readmission within 30 days after discharge. Karls et al. [26] reported a higher prevalence of malnutrition in pediatric scoliosis patients undergoing multiple surgeries, with malnourished individuals more likely to need debridement for postoperative wound infections. A multicenter study of 2799 AIS patients revealed that underweight patients had a higher complication rate (3.9% vs. 1.4%) compared with those with normal weight [27].

### 4.4 Factors Associated With Postoperative Complications and Clinical Implications

Previous studies have analyzed the influencing factors of complications after growing‐rod surgery for EOS, including initial surgical age, number of distractions, initial main Cobb angle, and excessive lordosis [28]. In this study, we further analyzed the risk factors for complications in EOS patients. We found distraction surgery ≥ 3 times and nutritional status as key predictors. In the conventional GEE model, malnutrition was identified as an independent risk factor for postoperative complications in EOS patients (OR = 3.701, 95% CI: 1.006–13.613). Although the preoperative main curve Cobb angle differed between the nutritional groups, additional multivariable GEE analysis incorporating Cobb angle showed that the association between height‐adjusted malnutrition and postoperative complications remained essentially unchanged, whereas Cobb angle itself was not significantly associated with complications (*p* = 0.763) (Supplement Table 1). However, because Cobb angle is directly incorporated into the Ylikoski height‐correction formula, height‐adjusted nutritional status is mathematically coupled with curve severity. Recent multicenter evidence also showed that both underweight and overweight/obesity were associated with greater initial curve magnitude and earlier curve progression in idiopathic EOS, further illustrating the close relationship between weight status and deformity severity [29]. In the postadjusted normal group, three procedures (7.5%) developed postoperative complications, all of which were wound infections. By contrast, among the 11 procedures (24%) with postoperative complications in the malnourished group, three (6.5%) had wound infections and eight (17.4%) experienced implant‐related complications. Additionally, one of these procedures had combined PJK as a concurrent complication. These findings suggest that malnutrition in EOS patients primarily increases the risk of implant‐related complications. Previous studies have reported that higher BMI is associated with increased implant‐related complications, potentially due to increased mechanical loading on spinal instrumentation [30]. In contrast, our findings suggest that malnutrition may represent another risk state, particularly in EOS patients, through mechanisms related to reduced muscle mass, limited posterior soft tissue support, and impaired biological capacity for maintaining spinal constructs. These findings indicate that abnormal nutritional status may influence implant outcomes through different biomechanical and biological pathways. Furthermore, malnutrition severely impairs growth and skeletal development, which may further contribute to the higher incidence of complications. Nevertheless, this finding requires further confirmation in larger, adequately powered studies.

Moreover, multiple distractions via the same incision elevate infection risk and impair skin healing, while repeated procedures may loosen screws, increasing screw pullout or rod breakage risks. This finding is consistent with previous reports, suggesting that limiting the number of distractions prior to final fusion may help reduce mechanical stress [10, 28, 31]. While advanced techniques such as MCGR have been developed to reduce the frequency of surgical interventions and associated risks, it is important to note that MCGR has not yet been introduced or gained widespread clinical availability in our country. Consequently, TGR remains the primary surgical option in our clinical practice. This regional technical constraint necessitates a focus on optimizing nutritional status to mitigate the higher complication risks inherent in multiple planned reoperations required by TGR.

### 4.5 Strengths and Limitations

This study has several limitations. First, as a retrospective, single‐center study, our sample size was relatively small. Because 53 patients underwent 86 surgeries, the data are naturally correlated. Although we used GEE to correct this “pseudoreplication” issue, selection bias may still exist. Second, only 14 procedures experienced postoperative complications, comprising 15 specific complication events. This limited the number of variables that could be included in the multivariable model and may have increased the risk of model instability. To evaluate the robustness of our findings, we performed a sensitivity analysis using a small‐sample correction for GEE variance estimation. Although the association between malnutrition and postoperative complications remained directionally consistent after conservative correction (OR = 4.21, 95% CI: 0.38–46.12), the confidence interval became substantially wider and statistical significance was attenuated. Therefore, the findings, including the relatively high AUC value of our model, should be interpreted cautiously and require validation in larger multicenter cohorts. Third, the Ylikoski formula was originally developed for idiopathic scoliosis and may not be perfectly accurate for all etiological types of EOS. Future multi‐center studies with larger cohorts are needed to confirm our findings. Fourth, the generalizability of our findings is further limited by the selected study population and surgical technique. Only patients with idiopathic or congenital EOS who underwent the initial growing‐rod procedure at 6–9 years of age were included, whereas patients with neuromuscular or syndromic scoliosis were excluded. In addition, all patients were treated with TGR. Therefore, the findings may not be directly applicable to other EOS subtypes, different age groups, or patients treated with MCGR or other growth‐friendly techniques. Fifth, the observed associations do not establish a mechanical or biological mechanism. Although GEE accounted for the within‐patient correlation among repeated procedures, previous complications were not modeled as a time‐varying covariate and may have influenced the planning, management, or risk of subsequent procedures. Therefore, a potential carryover effect from an earlier complication to a subsequent procedure cannot be excluded.

## 5 Conclusion

In EOS patients undergoing growing‐rod surgery, preoperative malnutrition and multiple surgeries (≥ 3) were associated with a significantly higher incidence of postoperative complications in the conventional GEE analysis. However, the association between height‐adjusted malnutrition and complications was not statistically significant after small‐sample correction. These findings should be interpreted cautiously and require confirmation in larger studies.

## Author Contributions

**Yangpu Zhang:** conceptualization, validation, visualization, software, writing – review and editing. **Haoshuang Geng:** formal analysis, visualization, writing – review and editing, writing – original draft. **Jianqiang Wang:** data curation, software, methodology, writing – original draft, writing – review and editing. **Yong Hai:** supervision, funding acquisition, conceptualization, methodology, investigation.

## Funding

This work was supported by the Clinical research incubation project, Beijing Chaoyang Hospital, Capital Medical University (CYFH202218).

## Ethics Statement

This study was performed in line with the principles of the Declaration of Helsinki. Approval was granted by the Ethics Committee of Chao‐Yang Hospital (Date: December 25, 2024/No. 2024‐KE‐964).

## Consent

Written informed consent was obtained from the parents.

## Conflicts of Interest

The authors declare no conflicts of interest.

## Supporting information


**Supplement Table 1.** Multivariate generalized estimating equation analysis of independent risk factors for postoperative complications.

## Data Availability

The data that support the findings of this study are available from the corresponding author upon reasonable request.
