# Supplementary material for: The Influence of Height‐Adjusted Nutritional Status Assessment on Complications Following Growing‐Rod Surgery for Early‐Onset Scoliosis
Source: Orthop Surg. 2026 Nov 6:10.1111/os.70461. Online ahead of print. doi: 10.1111/os.70461 (PMC13743319; doi:10.1111/os.70461)
Supplement: Supplementary file 1 [file OS-9999-0-s001.docx]

| **Supplement Table 1. Multivariate Generalized Estimating Equation Analysis of Independent Risk Factors for Postoperative Complications** | | | |
| --- | --- | --- | --- |
| Parameters | OR | 95%CI | P |
| Surgery frequency | 4.522 | 1.168-17.514 | 0.029 |
| Adjusted nutritional status | 3.943 | 1.054-14.761 | 0.041 |
| Main curve cobb angle | 1.007 | 0.964-1.051 | 0.763 |
